# Microneedles As a Delivery System for Gene Therapy

**DOI:** 10.3389/fphar.2016.00137

**Published:** 2016-05-26

**Authors:** Wei Chen, Hui Li, De Shi, Zhenguo Liu, Weien Yuan

**Affiliations:** ^1^Department of Neurology, Xinhua Hospital affiliated to the Medical School of Shanghai Jiao Tong UniversityShanghai, China; ^2^School of Pharmacy, Shanghai Jiao Tong UniversityShanghai, China

**Keywords:** micronnedles, gene, delivery, therapy, approaches

## Abstract

Gene delivery systems can be divided to two major types: vector-based (either viral vector or non-viral vector) and physical delivery technologies. Many physical carriers, such as electroporation, gene gun, ultrasound start to be proved to have the potential to enable gene therapy. A relatively new physical delivery technology for gene delivery consists of microneedles (MNs), which has been studied in many fields and for many molecule types and indications. Microneedles can penetrate the stratum corneum, which is the main barrier for drug delivery through the skin with ease of administration and without significant pain. Many different kinds of MNs, such as metal MNs, coated MNs, dissolving MNs have turned out to be promising in gene delivery. In this review, we discussed the potential as well as the challenges of utilizing MNs to deliver nucleic acids for gene therapy. We also proposed that a combination of MNs and other gene delivery approaches may lead to a better delivery system for gene therapy.

## Introduction

Gene therapy is a technique to transport genetic materials to a specific cell with the aim to correct or compensate for the genetic defects, thereby achieving the goal to treat diseases (Mulligan, 1993). With the development of molecular biology and biotechnology, we can replace the mutant gene in the diseased cells to treat genetic diseases, such as hemophilia, muscular dystrophy, cystic fibrosis (Mulligan, 1993). We can also treat genetic disorders by delivering genetic materials to targeted cells (Pack et al., 2005). The first gene therapy clinical trial started with the severe combined immunodeficiency (SCID) in 1990 (Blaese et al., 1995). Cavazzana-Calvo firstly reported the successful clinical case of gene therapy in April, 2000 (Cavazzana-Calvo et al., 2000). In July 2012, the European Medicines Agency recommended the approval of Glybera, which is a gene therapy for the treatment of lipoprotein lipase deficiency (LPLD) (Bryant et al., 2013; Watanabe et al., 2015).

There have been many clinical trials of gene therapy approved so far, but the success rate has been low (Kay et al., 2000; Khuri et al., 2000). On the other hand, the discovery of the phenomenon of RNA interference may open a new road for the gene therapy (Fire et al., 1998). Compared with the traditional gene therapy, siRNA can efficiently silence the diseased gene and knock down its function. The sequences of siRNAs can be rationally designed to target specific genes. The effects of siRNA can be quick and significant. All the recent progresses make gene therapy enter a new revolution. Recent therapeutic trials of siRNA have been carried out with macular degeneration, diabetic macular edema, solid tumors, respiratory diseases, syncytial virus, and human immunodeficiency viral infections, etc. (Novobrantseva et al., 2008; Castanotto and Rossi, 2009; Whitehead et al., 2009). However, Andrew Z. Fire, the Nobel Prize recipient for discovering RNA interference, said that the lack of efficient delivery systems of siRNA may be the next obstacle for gene therapy when he was awarded on the podium in 2006 (Fire, 2007).

The big challenge now is to find a safe and efficient delivery system to help genetic materials or siRNAs target to the specific cell. Microneedles (MNs), as a new transdermal delivery system, has the potential to greatly enhance the cutaneous delivery of low, medium, and high molecular weight therapeutic agents (Coulman et al., 2006). Recently, MNs has also been studied to deliver genetic materials for gene therapy (Niidome and Huang, 2002; McCaffrey et al., 2015). In this review, some accessible MNs-based methods for gene delivery with clinical potential will be reviewed and the future prospects of MNs-based delivery systems will also be discussed.

## The existing problems of gene delivery

In general, the gene delivery systems have been developed into the following three major types: viral vectors, non-viral vectors, and physical delivery carriers. The related studies have proved their effectiveness in delivering genetic materials or siRNAs. But many critical issues still remained to be solved.

Viral vectors use the natural ability of virus to transport the genetic materials into infected cells. The construction of viral vectors retains the necessary components of the genetic material and knocks down the pathogenic components (McCaffrey et al., 2016). It has been widely applied in clinical trials of gene therapy for retrovirus, lenti virus, adeno-associated virus, and adenovirus, etc (Mountain, 2000; Cots et al., 2013; Kotterman et al., 2015). However, the gene delivery vectors derived from viruses have the potential to be pathogenic because of their potential tumorigenicity and immunogenicity. Moreover, the potential for vector integration and insertional mutagenesis also limits the use of viral vectors (Lentz et al., 2012). In addition, the costly scale-up production, insufficient capacity for cell targeting and low level of transgene expression remind us to find a more safe and effective vector for therapeutic gene delivery (Weinberg et al., 2013).

Non-viral vectors, another kind of vectors that can protect the nucleic acids from degradation and assist cellular entry, can be further divided into lipid cations, cationic polyplexes and others (Guo et al., 2013; Noori-Zadeh et al., 2014; Yin et al., 2014). Lipid cations, or liposome, incorporate, and compact negatively charged DNA into nanoparticles. They can be up-taken into the cytoplasm by endocytosis or membrane fusion. The delivery efficiency of lipid cations is quite high, but lipid cations may cause undesirable membrane destabilization and cytotoxicity. In addition, the unstable batch production makes it hard to be widely applied (Koirala et al., 2013). Cationic polymers, including polyethyleneimine (PEI), poly-L-lysine (PLL), are polymers with cationic groups with positive charge that can compact nucleic acids, including primary amine, secondary amine, tertiary amine, and quaternary amine, etc. Because of the flexibility of chemical structure, cationic polymers-based polyplexes have many possibilities to deliver nucleic acids and cause less cytotoxicity (Noori-Zadeh et al., 2014). However, limited transfection efficiency and the relatively low ability to bind to nucleic acids impeded its clinical application (Xun et al., 2014). Others, like chitosan, modified cyclodextrins (CDs), PLGA, nanobubbles, cell penetration enhancer peptides (Hsu and Mitragotri, 2011; Cavalli et al., 2013; Favaro et al., 2014; Lai, 2014) can complex nucleic acids and form nanoparticles and transfect a wide range of cell types. However, for these carriers, the issues of efficient loading and the optimal duration of therapeutic effects need to be carefully addressed.

Physical delivery carriers depend on physical methods to deliver nucleic acids into cells, such as electroporation, gene gun, ultrasound, hydrodynamics high pressure injection and microneedles (Somiari et al., 2000; Newman et al., 2001; McCaffrey et al., 2016). The delivery process is simple, but the nucleic acids can be degraded by nucleases easily, so in general the nucleic acids need to be chemically modified to improve its stability and biological activity.

## The advantages of microneedles for gene therapy

**Figure 1:**
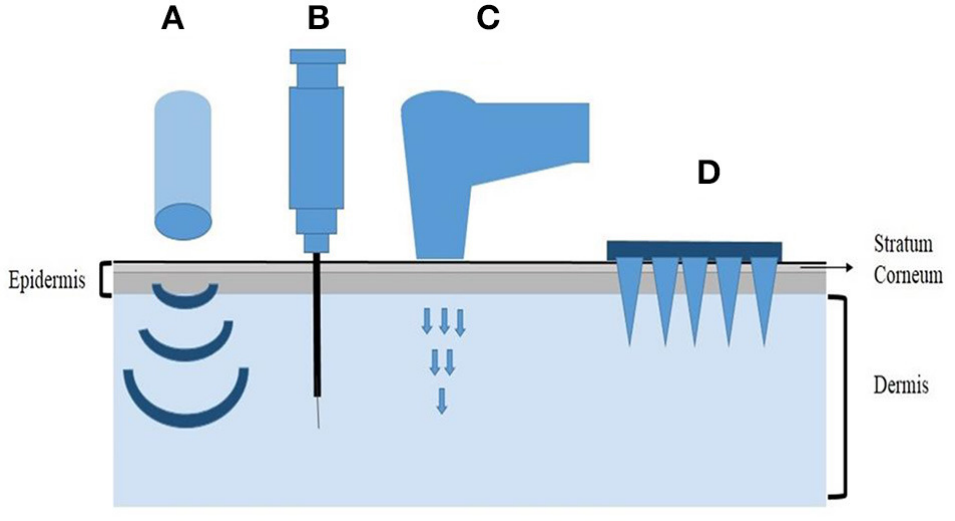
**Representative physical methods for transdermal delivery. (A)** Ultrasound methods, **(B)** Intradermal injection, **(C)** Gene gun, **(D)** Microneedles.

Four major physical methods have been developed so far for the transdermal delivery of therapeutics including ultrasound methods, intradermal injection, gene gun and microneedles (Figure 1). Microneedles (MNs), as a minimally invasive drug delivery system, can deliver both low-molecular weight and high-molecular weight agents, including the nucleic acids into the systemic circulation by penetrating the stratum corneum (SC), which is the main barrier for intradermal drug delivery. Microneedles are shaped with arrays of needles ranging from 25 to 2000μm in height (Donnelly et al., 2010). The needles have different tip shapes and tip intervals, being attached to a base support. The dimension of microneedles is within the micron range, but greater than the size of their cargos, so it will be easy for macromolecules, even drug-excipient complexes or nanoparticles to get through the micro-channels in the microneedles. The application of microneedles is pain-free and patient-friendly, which greatly improves the patient compliance. Since it is easy to use, microneedles also have the potential for self-administration. In addition, the low production cost makes microneedles a promising drug delivery system with marketing potential as well (Coulman et al., 2006).

The fabrication of microneedles usually involves many materials, including stainless steel, ceramics, dextrin, polymers and glass (Frazier, 2003; Ito et al., 2006; Ovsianikov et al., 2007; Jiang et al., 2009). Moreover, different fabrication methods have been used to construct microneedles, including chemical isotropic etching method, micro-molding method, the surface/ultrafine processing method, lithography—electroforming—replication method. Laser-etching prepared microneedles have also been reported Trichur et al., 2002; Moon and Lee, 2005). Based on their properties, microneedles can be divided into several types: solid microneedles, holliow microneedles, dissolving microneedles, and hydrogel microneedles (Liu et al., 2014).

Now microneedles have been used for the transdermal delivery of a broad range of drugs, such as small molecular weight drugs, oligonucleotides, DNA, peptides, proteins, and inactivated viruses. Moreover, extensive experiments with influenza, Calmette–Guérin (BCG), and other vaccines have shown that vaccine delivery into the skin is also promising (Gonzalez-Gonzalez et al., 2009; Haq et al., 2009; Kim Y. C. et al., 2012a; van der Maaden et al., 2012). Recently, microneedles combined with siRNA have been applied as gene therapy. As reported, it can efficiently and reproducibly deliver nucleic acids to the skin for the treatment of genetic skin disorders, cancers, wounds, and hyper proliferative diseases (McLean and Moore, 2011).

## The solid microneedles

The solid microneedles are usually made of metals and silicon using dry or wet etching process. By using potassium hydroxide (KOH) solution, the part of silicon needles that are not covered by chromium model can be etched into required shape (Lin and Pisano, 1999). The solid microneedles usually need a two-step application. First, it penetrates the SC to form some transient microchannels. Then the drug solution, or gel, cream, ointment is applied as a form of a patch on the area of skin. Guang Yan et al. (Birchall et al., 2005) developed a motorized metal-based microneedle device for transdermal delivery of plasmid DNA that encoding enhanced green fluorescent protein and firefly luciferase (pEGFP-Luc). The authors found that high gene expression can be obtained and the skin would have minimal damage with the device. In their study, the authors also compared the different methods for the delivery of DNA into the skin, including passive diffusion method, intradermal injection method and motorized microneedle device method for delivering DNA solution. Motorized microneedle device turned out to be more advantageous than the other two methods in equivalent conditions. And the result showed that pretreatment of skin with microneedles followed by application of DNA solution is less effective than the application of DNA solution at first. The gene expression of latter method is 87 times higher than the former one. In addition, Kumar et al. (2012) used Dermaroller® microneedle roller, which is also a kind of solid microneedles made into roller to pretreat the mice skin. Then the plasmid DNA coated on the surface of cationic PLGA nanoparticles are applied onto the microneedle-treated area. The results showed that microneedle-mediated transcutaneous immunization with plasmid DNA carried by the nanoparticles induced a stronger immune response than with the plasmid DNA alone. So the solid microneedles, used as a pretreatment to deliver nucleic acids subcutaneously appears to be promising.

## The coated solid microneedles

The coated solid microneedles avoid a two-step application, which uses a dip-coating method to coat the proteins, DNA, viruses or microparticles onto the surface of the needles. The material used to fabricate the microneedles can be metal, silicon or even polymer materials. Pearton et al. (2012) fabricated the steel microneedles into 75 μm thick using an infrared laser. An optimized dip-coating process was used to coat the pDNA onto the MNs, of which the loading capacity is up to 100 μg of pDNA per 5-microneedle array. They believed that suitable DNA loading, efficient and reproducible skin puncture and rapid *in vivo* dissolution of pDNA at the treated site determine the efficiency of gene expression from coated microneedles. Chong et al. (2013) used wire electrical discharge machining (EDM) to fabricate the stainless steel microneedle devices which contained either 5 or 10 needles of 700 μm length and 200 μm base width. A coating reservoir was used to load BLOCK-iT™ Alexa 647 fluorescent siRNA into a pipette tip and microneedles. The theoretical loading onto each microneedle device was 35 μg siRNA. The fluorescence image showed that the coating of 0.1 μg fluorescent siRNA on the surface of a single steel microneedle was removed from the surface after a 10 min insertion, which verified the distribution of siRNA. For *in vivo* studies, the microneedles were coated with self-delivery siRNA targeting the reporter genes (luciferase/GFP). The intravital imaging of reporter gene expression and the quantification of reporter mRNA confirmed the functionality of gene silencing following microneedle delivery. The results demonstrated that coated solid MNs have the potential to deliver nucleic acids *in vivo*. The further study should focus on the delivery of larger doses of therapeutic siRNAs.

## The dissolving microneedles

**Figure 2:**
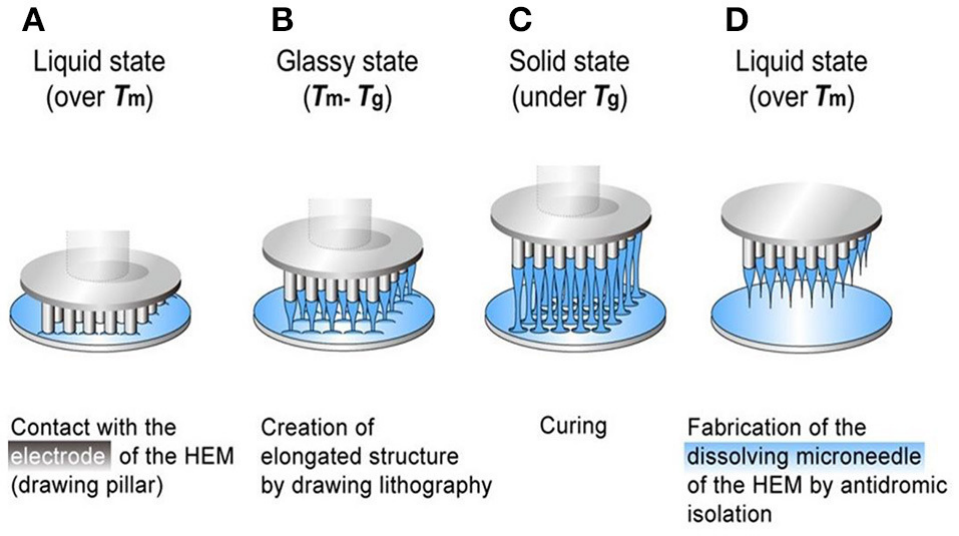
**The monolithic fabrication of a HEM by drawing lithography with antidromic isolation. (A)** Liquid maltose was coated on a planar surface, and contacted with the 5 × 5 array electrodes of the HEMs as a drawing pillar. **(B)** The glassy maltose between Tm and Tg was elongated by drawing of electrodes. **(C)** After lowering the temperature to sub-Tg, the elongated 3D structures were cured to a solid state. **(D)** The coating surface was melted at a temperature greater than Tm to isolate elongated 3D structures from 2D coating surface, and this antidromic isolation fabricated dissolving microneedles of the HEMs. Adapted with permission from (Lee et al., 2011).

Dissolving microneedles are always made of biodegradable polymers, which can dissolve or degrade within the skin. As a result, there is no need to remove the MNs from patients' skin. Compared with coated microneedles, dissolving microneedles have bigger loading capacity. The drugs loaded into the needles can quickly release after the insertion into the skin (Donnelly, 2011). Lee et al. (2011) used a hybrid electro-microneedle (HEM) to achieve a safe and high-capacity gene transfer. The monolithic fabrication process of the HEM is shown in Figure 2. The HEM is a monolithic hybrid assembly of a dissolving microneedle and an electrode. The electrode was used as a drawing pillar to elongate dissolving microneedles from 2D glassy maltose. The needles are separated by antidromic isolation in the melting process. The dissolving microneedles have an ultra-sharp tip diameter of 5 μm and a length of 400 μm. The cutaneous permeation and release tests showed that the microneedles of the HEM dissolved completely in 20 min after insertion into the skin. Then the electrode applied electric pulse to facilitate intracellular transfection of nucleic acids into cells at the release site. Compared with the negative controls (pCMV-GLuc with dissolving microneedles alone and the control PCI plasmid with HEM), the bioluminescence intensity achieved by pCMV-GLuc transfer with the HEM was significantly higher after 8 to 15 days. The regression of subcutaneous B16F10 by cutaneous p2CMVmIL-12 transfer using a HEM also lead to a longer survival time of 45 days for the tested mice.

Gonzalez-Gonzalez et al. (2010) applied a PAD (Protrusion Array Device) loaded with a fluorescently tagged siRNA mimic (siGLO Red) onto the mice footpad. They fabricated the dissolving microneedles under a controlled airflow. A pin template contacting with a thin film of PVA solution was withdrawn to form a fiber-like structure, which then was trimmed to a uniform height with sharp tips. The distribution and silencing of CBL/hMGFP reporter gene demonstrated that endogenously expressing genes can be reduced 25–50% by Accell siRNAs delivered by PADs. And they proposed a combinatorial approach of PAD and Accell may result in greater efficiency to knock down the target gene.

**Figure 3:**
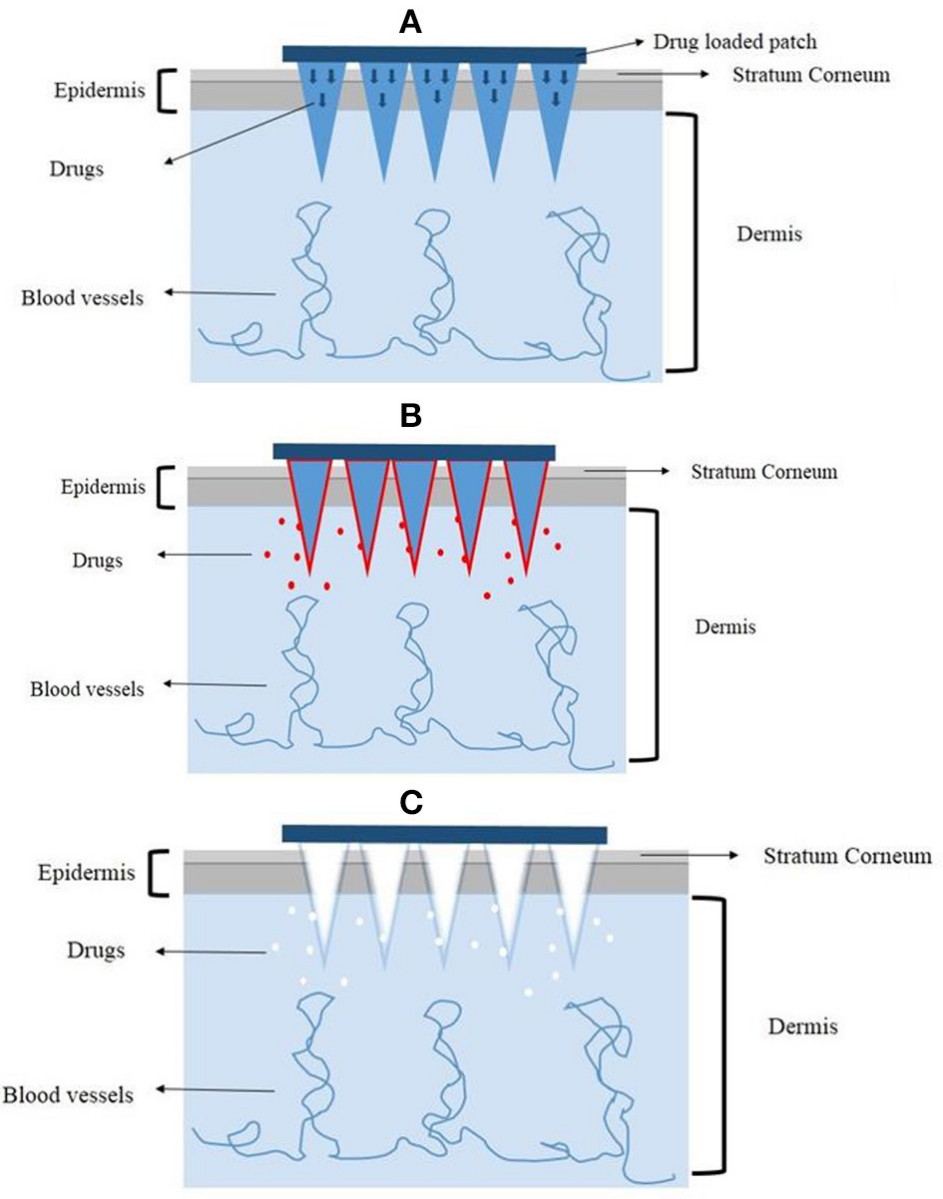
**The mechanisms of different microneedles to deliver drugs**. **(A)** The solid microneedles. **(B)** The coated microneedles. **(C)** The dissolving microneedles.

Lara et al. (2012) also used the PAD to load CD44 sd-siRNA both *in vitro* and *in vivo*. The treatment with CD44 sd-siRNA decreased CD44 mRNA levels, resulting in a reduction of the target protein as confirmed by immunodetection. The results demonstrated that administration of dissolvable microneedle arrays, loaded with sd-siRNA can reduce expression of a targeted endogenous gene in a human skin xenograft model. Based on these favorable results, the dissolving microneedles could be a good alternative to deliver genetic materials. A graphic illustration of the mechanisms of different microneedles for therapeutics delivery is shown in Figure 3.

## The existing problems of MNs to be used for gene therapy

Although the microneedles have been verified as an effective and potentially useful way to deliver siRNA or other nucleic acids into the skin, many problems still remained to be addressed.

The motorized solid microneedle device utilized by Birchall et al. (2005) may have the concerns of costly fabrication and the two-step application still increases the inconvenience of self-administration. In addition, the treated skin needed to be punctured for sufficient long duration to get the high gene expression in the skin. During the application, the motorized microneedle device could cause some undesirable damage to the skin, though the study claims to only cause the minimal damage. Moreover, the breakdown of microneedles in the skin may be another potential safety problem for solid microneedles.

The coating of coated microneedles could be restricted to the tips of microneedles. Devices used to enhance the depth of skin penetration may also be a potential issue (Coulman et al., 2011). The coating formulations and procedures may limit the penetration of skin and efficient drug deposition at the targeted site, since the viscosity and surface tension of the formulation could affect the uniform coating of the microneedles (Chong et al., 2013). In addition, the coating capacity could be another problem. Pearton et al. (2012) reported the fabrication of the coated microneedles with high dose-loading of pDNA (up to 100 μg of pDNA per 5-microneedle array). However, compared with other kinds of microneedles, the loading capacity of the coated microneedles still needs to be improved.

The dissolving microneedles is composed of biodegradable polymers, which has the potential issue of biocompatibility *in vivo*. Since the dissolving microneedles almost dissolve completely after the insertion into the skin, we should pay attention to the safety and biocompatibility of these dissolving materials and their degradation products. Moreover, the release kinetics of the dissolving microneedles depends on the internal structure of the polymer material, which could be an obstacle for the delivery into the skin. In addition, some microneedle fabrication procedures involve high polymer-melting temperatures, some are even above 135°C, which will be damaging to some temperature-sensitive drugs, such as proteins and nucleic acids. Another issue is that the loading amount of drugs may also affect the needle mechanical properties when the main matrix is PLGA or carboxymethyl cellulose. For example, Kim M. et al. (2012b) found that microneedles with 10% drug loading could not keep the mechanical strength to penetrate the skin. In addition, for therapeutic nucleic acids, the intracellular barriers including endosome escape, releasing from carriers, and entering into nucleus could be additional challenges. Combining MNs and nanoparticle-based formulations could be a potentially useful approach to overcome these intracellular barriers (Kumar et al., 2012; McCaffrey et al., 2016).

Because of the properties of MNs, the application site is limited to some certain area on the body, such as arms, hands, abdomen where the epidermis and SC are easier for MNs to penetrate. As reported, the dissolving microneedles dissolved completely in 20 min (Lee et al., 2011), so the duration time for the application is also critical.

No matter solid microneedles, coated microneedles, dissolving microneedles, or hollow microneedles, the MNs are used as a physical delivery method. For gene therapy, the cargo nucleic acids also need to be improved. MNs facilitating the delivery of genetic material alone may not be better than intradermal injection, but the combination of MNs and electroporation proved to be more effective than injection alone, which gives us a thought of the combined use of different delivery techniques. While the other physical delivery carriers have some shortages such as damages to the skin, the costly fabrication and patient-unfriendly etc., MNs behaves well at those aspects, and the electrically controlled pulse may further improve its efficiency. And some bioactive components can also be added into the formulation of dissolving microneedles to enhance its effects. Moreover, the advantage of pain-free application makes microneedles to act as an optimal strategy for DNA vaccination. MNs could relieve the patients from the suffering of multiple injections from days to weeks to develop immunization.

In conclusion, the further development of microneedles for gene therapy depends not only on the improved fabrication of MNs, such as the structures, the mechanical properties and the formulations etc., but also on the combined use of different delivery techniques to achieve optimal therapeutic outcomes.

## Author contributions

WY and ZL conceived and participated in its design, searched databases, extracted and assessed studies and helped to draft the manuscript. WC, HL, and DS participated in the conceptualization and design of the experiment, data extraction and analysis, WC, and HL wrote the manuscript. All authors read and approved the final manuscript.

### Conflict of interest statement

The authors declare that the research was conducted in the absence of any commercial or financial relationships that could be construed as a potential conflict of interest.
